# Autonomies in Interaction: Dimensions of Patient Autonomy and Non-adherence to Treatment

**DOI:** 10.3389/fpsyg.2019.01857

**Published:** 2019-08-14

**Authors:** Ion Arrieta Valero

**Affiliations:** Department of Philosophy, ETICOP-IT: Ethics Training in Communities of Practice - Ikerketa Taldea (Research Group), IAS-Research Center for Life, Mind and Society, University of the Basque Country (UPV/EHU), Donostia-San Sebastián, Spain

**Keywords:** patient autonomy, decisional autonomy, executive autonomy, narrative autonomy, capacity, identity, adherence to treatment, psoriasis

## Abstract

In recent years, several studies have advocated the need to expand the concept of patient autonomy beyond the capacity to deliberate and make decisions regarding a specific medical intervention or treatment (*decision-making* or *decisional autonomy*). Arguing along the same lines, this paper proposes a multidimensional concept of patient autonomy (decisional, executive, functional, informative, and narrative) and argues that determining the specific aspect of autonomy affected is the first step toward protecting or promoting (and respecting) patient autonomy. These different manifestations of autonomy are not mutually dependent; there may be patients who have problems in one dimension, while at the same time being fully autonomous in others. Nevertheless, a close interaction has been observed between the various dimensions, and indeed, a phenomenological analysis shows that damage to or a reduction in one aspect of people’s capacity for self-government generally affects other aspects of their autonomy, which in turn disrupts their identity and the way in which they see themselves and are seen by others. In this paper, I shall examine some of these interactions and show how they may lie at the heart of the problem of poor treatment adherence in many patients with chronic ailments (where adherence is defined as being the extent to which a patient’s behavior over time coincides with the recommendations made by and agreed with their health professional). One example given is that of psoriasis, a chronic skin disease with a very poor adherence record. In Spain, it is calculated that 85% of patients diagnosed with mild to moderate psoriasis fail to comply properly with their treatment, and figures from other parts of the world are similar. Although there are many possible causes for non-adherence among psoriasis patients, assessing their decisional, executive, and narrative capacities and taking appropriate action based on the results may help increase adherence rates.

## Introduction

The debate on patient autonomy has been a central feature in the development of bioethics and, more specifically, clinical ethics. In one of the most influential works written on the subject, Beauchamp and Childress (1979/2001, p. 58) state that all theories of autonomy agree that two conditions are essential for autonomy: *liberty* (independence for controlling influences) and *agency* (capacity for intentional action). However, medical ethics has largely been more concerned with the regulatory dimension, i.e., the *principle of respect for autonomy*, than with the theories of autonomy underpinning that principle (Arrieta, 2016). It has emphasized the first element (the liberty that must be afforded subjects to choose and act) and neglected the second (their capacity to implement the options they freely choose). Much of the clinical literature centers more on legal aspects of how to protect a right than on the personal or subjective aspects underlying patients’ capacity to decide autonomously (Casado and Etxeberria, 2014, p. 36).

The prevailing perspective of autonomy is strongly influenced by legal and juridical constructs designed to protect the right that citizens enjoy under normal circumstances to run their own lives (Gracia, 1989/2008, 2012; Tauber, 2005, 2011; Arrieta, 2012; Arrieta and Casado, 2014), such as those proposed by Frankfurt (1971), Dworkin (1988), and Christman (1989). These definitions of autonomy were not developed specifically for individuals with a disease or disability, but rather for healthy agents and citizens. These models of autonomy have been influential in discussions related to free will (Müller and Walter, 2010, pp. 206–207) but have also become the standard model of autonomy used in bioethics (Felsen and Reiner, 2011).

Agich (2007) writes that chronic care has generally been of only peripheral interest in bioethics. In the final decades of the twentieth century, for reasons of pragmatic necessity and operativity, work in the field concentrated mainly on the fast and urgent decisions that have to be made in tertiary healthcare, with immediate and sometimes dramatic, life-or-death, consequences. Because the concept of patient autonomy has been developed within the context of acute care, it rightly centers on *decisional (or decision-making) autonomy*, i.e., the patient’s capacity to understand information and to make voluntary decisions (Naik et al., 2009). The most widespread notion of autonomy is identified with the freedom of choice of someone who is rational and capable of making decisions (Cassell, 2010). In the literature on bioethics, there is a constant tendency to equate autonomy with autonomous decision-making (see seminal works on medical ethics such as Reich, 1978; Beauchamp and Childress, 1979/2001; Jonsen et al., 1982), and the greatest autonomy-related problems are generally linked to issues such as informed consent, decision-making capacity, and surrogate decision-making in the case of people who have been declared mentally incompetence (Agich, 2007, pp. 74–75).

The nature of autonomy varies depending on the social context in which the concept is applied (Anderson, 2013, 2014a,b). In acute care situations, it is quite appropriate to view autonomy almost exclusively in decisional terms, for the purposes of accepting or rejecting a specific therapeutic decision. This decision-making process can be approached in a similar way to that habitually exercised by healthy and able-bodied individuals, and legal or juridical notions of autonomy may therefore be both appropriate and useful. However, these models of autonomy are much less satisfactory when it comes to situations of chronic disease and primary medicine, where interaction between professionals and patients is notably different and specific decisions are probably less important than the continued maintenance of the relationship itself. As well as strictly medical issues and respect for freedom of choice, greater attention should be paid to the particularities of patients or people in need of healthcare, i.e., the biological, psychological, and social aspects that enable them to be autonomous. In many such cases, especially in situations of chronic fatal illness or degenerative diseases, any autonomy that does exist is precarious and in decline and therefore considerably removed from the “autonomy yes/autonomy no” way in which it is presented in decisional ethics. In situations of illness or weak or precarious health, autonomy has a different meaning than in other areas (such as the legal context) for the simple reason that it is diminished or compromised. Because autonomy can only be respected if it exists (Matthews, 2007, p. 129), before respecting autonomy, healthcare workers must first seek to restore it (Arrieta, 2012, p. 28). Many authors therefore consider it paradoxical to view respect for autonomy as the overriding rule in medical ethics, given that in many cases, there is very little autonomy to be respected (Kittay, 2007; Nys et al., 2007).

Viewing decision-making capacity as the only feature of autonomy means ignoring many of its other manifestations. For this reason, it is necessary to “decentralize autonomy” (Meyers, 2005). While capacities for critical reflection or rational decision-making are essential in managing autonomous conduct, they belong to only one of the registers through which human autonomy emerges. In recent years, several authors (Casado, 2009; Naik et al., 2009; Seoane, 2010, 2013; Arrieta and Casado, 2014; Casado and Etxeberria, 2014; Arrieta, 2016) have advocated expanding the concept of patient autonomy to include not only patients’ ability to make free and therapeutically informed decisions (the decisional dimension) but also their capacity to plan, sequence, and perform tasks related to the management of their chronic diseases, i.e., to adhere to the chosen therapeutic plan (the executive dimension). Other areas to be included are the ability to perform the basic vital functions and tasks that can be carried out by a statistical majority of people (the functional dimension); to have control information on their situation in the manner of their choosing (informative dimension); and to retain, understand, and communicate to others, in a sufficiently coherent and understandable manner, the main identitary aspects that have characterized them during their lives (the narrative dimension).

This paper is based on the premise that knowing which aspect or aspects of autonomy are affected is a necessary prerequisite for protecting or promoting (and respecting) patient autonomy. Different ailments or circumstances (a medullar injury, depression, poor management of information, etc.) involve the impairment of one or more different manifestations of an individual’s autonomy, and each one should therefore be studied separately. One of the requirements for a good understanding of a patient’s situation and the provision of good medical care is therefore to identify which dimensions are compromised or damaged. This leads us to pose a number of research questions: How are these dimensions related? Do they interact? If so, how? And what effects do they have on patients’ identity and medical and care processes?

The different manifestations of patient autonomy are not necessarily related or mutually dependent. Patients may show a deficiency in one manifestation, while being fully autonomous in others. Nonetheless, a close interaction can be observed between the different dimensions, and a major deficit in one capacity can cause a limitation in others. This combination of capacities directly impacts peoples’ identity, i.e., how they see themselves and how others see them. This paper offers some examples of these interactions and seeks to show how they may lie at the root of non-adherence issues in many patients with chronic ailments. Although there are many reasons for non-adherence (World Health Organization, 2016), my hypothesis is that appraising patients’ decisional, executive, and narrative capacities and acting accordingly may contribute to improving the situation. To test this hypothesis, I take a chronic skin disease, psoriasis, which presents very low levels of adherence to treatment.

## Adherence to Treatment

The management and treatment of chronic diseases is one of the most pressing challenges facing societies with an aging population, as is the case in Europe. As well as ruining the lives of millions of people, chronic diseases can be extraordinarily costly for society when not treated successfully. Non-adherence is a global phenomenon with serious consequences: loss of control over the disease, high costs for the health system due to an increased level of hospital admissions and readmissions, frustration among healthcare workers, reduced quality of life, high degree of family, and social attrition, etc. (Rojas Marcos, 2012). Adherence to long-term therapy for chronic illnesses in developed countries averages 50%. In developing countries, the rates are even lower. Increasing the effectiveness of adherence interventions may have a far greater impact on the health of the population than any improvement in specific medical treatments (World Health Organization, 2003, p. XIII). Understanding the causes of low adherence to treatment and developing strategies for neutralizing them would therefore provide enormous benefits to patients, medical professionals, and society at large.

I define adherence as being the degree to which patients’ behavior over time (taking a drug, following a diet, altering habits and lifestyle) coincides with the recommendations agreed between them and their healthcare professional. In the area of treatment, non-adherence may be either primary (failing to redeem the prescription at the pharmacy) or secondary (forgetting to take the drugs, prematurely discontinuing treatment, taking incorrect doses, changing dosing intervals, etc.) (Puig et al., 2013, p. 493).

Over the past few decades, the development of approaches aimed at ensuring that patients continue therapy for chronic conditions over long periods of time has gone through several phases. Initially the patient was thought to be the source of the “compliance problem.” Compliance is the fulfillment by a patient of a caregiver’s prescribed course of treatment. The term “adherence” has been proposed as an alternative to compliance and is growing in popularity. The word adherence is now preferred by many healthcare providers because “compliance” suggests that the patient is passively following the doctor’s orders and that the treatment plan is not based on a therapeutic alliance established between patient and physician. Furthermore, the idea of compliance is too closely associated with blame, on the part of either the providers or the patients, and the concept of adherence is a better way of embracing the dynamic and complex changes required of many individuals over long periods in cases of chronic disease. While the term “compliance” is seen as being overly normative and focuses exclusively on the patient’s behavior, the term “adherence” involves an assumption of shared responsibilities. Adherence is a complex behavioral process determined by various interacting factors, including the specific characteristics of the patient and the nature of the disease and its treatment but also the patient’s environment (operation of sanitary equipment, characteristics of the health system, social support, accessibility to health services, etc.). Physicians may contribute to poor adherence among patients by prescribing complex regimens, failing to adequately explain the benefits and side effects of a medication, not taking the patient’s lifestyle or the cost of the medication into consideration, and having a poor therapeutic relationship with their patients. Practitioners should always be alert for poor adherence and may mitigate the problem by emphasizing the value of a patient’s regimen, making it simple, and customizing it to the patient’s lifestyle (Vermeire et al., 2001; Osterberg and Blaschke, 2005; Barr, 2011).

Most of the studies conducted to date on treatment adherence relate to chronic diseases which involve a high cost for the patient, the healthcare industry and, by extension, the government also. Chronic diseases such as asthma, diabetes, hypertension, and HIV infection, or addictions such as smoking, have traditionally been cited in the literature as examples of challenges to adherence (World Health Organization, 2003; Osterberg and Blaschke, 2005). However, our knowledge of adherence levels in topical medication is limited. The reason may be the route of administration or the fact that, in many cases, the condition is not life-threatening (Peralta and Carbajal, 2008). Although many different methods are available to measure medication adherence, the lack of a gold standard for doing so in cases of topical therapy continues to pose challenges (Feldman et al., 2008). There is a need for improved quality of research and reporting in this area (Thorneloe et al., 2012).

## Psoriasis

Psoriasis is an inflammatory disease of the skin (and occasionally of the joints) which causes thick red patches or plaques of skin, covered with silvery scales. It is caused by abnormally rapid renewal of skin cells (whereas healthy skin cells are replaced every 28–30 days, among patients with psoriasis, the process takes 4–6 days). It is a non-contagious disease, with a certain genetic predisposition, although its exact etiology is largely unknown. Psoriasis develops erratically and unpredictably, with disease-free periods alternating with affected periods which may vary greatly in duration and intensity. Generally speaking, however, psoriasis is a chronic condition. It has been calculated that up to 80% of those affected suffer from the disease throughout their lives, either intermittently or continuously, with adverse emotional or psychological circumstances (bereavement, depression, periods of stress, etc.) sometimes acting as triggers or aggravators.

The negative impact of psoriasis on people’s lives can be immense. Psoriasis affects people of all ages and in all countries. The prevalence of psoriasis in countries ranges between 0.09 and 11.43%, making psoriasis a serious global problem with at least 100 million individuals affected worldwide (World Health Organization, 2016, p. 1). Psoriasis is one of the most frequent reasons for consulting a dermatologist (Puig et al., 2013) and one of the chronic diseases with the lowest adherence rates among patients. After years of work by a large number of patients’ organizations, in 2014, the World Health Organization passed a resolution recognizing psoriasis as “a chronic, non-communicable, painful, disfiguring, and disabling disease for which there is no cure”. This initiative turns the spotlight on the pathology, calling on member states to promote more research and to implement effective strategies to improve treatment, as well as encouraging them to engage further in advocacy efforts to raise awareness regarding the disease and to fight the stigmatization experienced by sufferers (World Health Organization, 2014, 2016).

Perhaps, the great therapeutic deficit with regard to psoriasis is that most of those affected either do not follow or incorrectly follow the treatment agreed upon with their doctor. In Spain, it has been calculated that 85% of patients diagnosed with psoriasis do not properly comply with treatment (Rojas Marcos, 2012); these figures are similar to those for other parts of the world (Kuehl and Shear, 2018). Patients with the lowest rates of adherence include those with mild to moderate psoriasis (i.e., affecting between 3 and 10% of the total body surface), who are generally prescribed topical treatment (creams, ointments, gels, etc.). For approximately 70% of patients, such therapy is their only option (Puig et al., 2013; Schaarschmidt et al., 2013; Kuehl and Shear, 2018). Topical therapy remains a pillar of psoriasis management, and adherence to treatment is a determining factor in ensuring efficacy. However, numerous studies specifically indicate that many patients with psoriasis consider the topical treatment to be one of the most negative aspects of the disease (Feldman et al., 2008; Rojas Marcos, 2012; Puig et al., 2013; Choi et al., 2017). The treatment must be applied correctly and on a continuous basis. It requires time, discipline, and constancy, and the results are not always evident or may be unsatisfactory, causing patient frustration and apathy. As a consequence, most patients either fail to apply the treatment properly or give up on it. It is estimated that nearly 50% of patients with psoriasis do not even purchase the prescribed product, and of those who do, up to 70% do not use their medication as per the instructions, which are often inadequate, confusing, or difficult to follow (Puig et al., 2013).

## The Five Dimensions of Patient Autonomy

Perhaps, because the concept originally stemmed from the legal tradition (Gracia, 1989/2008; Tauber, 2005, 2011), and because it initially centered on tertiary or emergency medicine (Agich, 2007; Naik et al., 2009), the prevailing clinical literature has tended to reduce patient autonomy to decision-making. The working assumption is that patients are autonomous if they show the capacity to make informed decisions. The obligation of healthcare workers is therefore to check that this capacity has not been diminished, either by the disease or any other circumstance. They must do everything possible to ensure that patients (or their representatives in case of incompetence) understand all issues related to their clinical status; inform them of the possible courses of therapeutic action available to them and make sure that they are acting of their own volition and not under any external duress. If so, patients are considered to be autonomous, and by extension competent to make decisions related to their bodies or health, and it is the practitioners’ duty to accept and respect their decisions.

*Decisional autonomy* refers to patients’ freedom of choice, in other words, their capacity to deliberate and decide on a course of action from among a suitable range of useful options (Seoane, 2013). This autonomy is exercised in a communicative process between the medical practitioner and the patient, subject essentially to three requirements: patients must (1) act voluntarily, i.e., with no external duress, (2) have sufficient information regarding the decision they are going to make (i.e., the aim of the decision, any risks and benefits and possible alternatives), and (3) have the capacity, i.e., possess a series of psychological (cognitive, volitional, and affective) capabilities, to be able to know, appraise, and manage this information properly, to make a decision and to express it (Simón, 2008, p. 327; Arrieta, 2016).

Decisional autonomy was the first dimension to be addressed and consolidated. It is also the most ethically and legally developed, based on the theory of informed consent (Seoane, 2013, p. 30). However, autonomy consists of much more than just the right to informed consent (which in many cases involves no more than asking a patient to sign a document they do not understand) and decision-making. Reducing autonomy in this way hampers the work of professionals and carers and can often create an atmosphere of mistrust amongst the different actors involved in the care relationship (Arrieta and Casado, 2014). Since it mainly affects the defense of users’ rights, the issue of autonomy is restricted to its legal dimension (Gracia, 2012) and to informed consent (Nys et al., 2007; Puyol, 2012). Subjective personal aspects are neglected (Casado and Etxeberria, 2014), turning the patient into a “thing with rights”, forced to make decisions while their autonomy is ignored (Tauber, 2005, p. 17).

Moreover, the individualist perspective of much of the bioethical discourse does not fit well when applied to chronic disease and primary and family care. Concepts such as patients’ rights and autonomy need to be reviewed in this context. Here a relational, narrative, and participatory model of autonomy, grounded in the specific physiological and psychological circumstances of each patient, is more appropriate. Work carried out with notions such as disability and dependence has shown that it is helpful to distinguish between the capacity to make decisions and the possibility of putting those decisions into practice (Seoane, 2010, p. 64). Authors from fields such as “Disability Studies” and “Independent Living Movements” have worked extensively with the notion of *functional autonomy*, i.e., patients’ capacity to perform the basic activities of daily living and to individually undertake tasks that a statistical majority of people normally perform (such as eating, seeing, walking, understanding complex situations, etc.). A number of different measures or indicators of an individual’s functional capacity are now available. They include some universal and comprehensive examples, such as those contained in the International Classification of Functioning, Disability and Health (ICF) (World Health Organization, 2001) and the Convention on the Rights of Persons with Disabilities (CRPD) (United Nations, 2006) and also more specific ones which measure and assess a concrete function.

The problem with these and other classifications is that they cannot help being somewhat arbitrary. The standard used to evaluate these functions or structures is generally “the statistical norm for humans” (World Health Organization, 2001), and it is not easy to establish a statistical norm for each human activity or to measure the degree of deficit or deviation with regard to it. Moreover, it is tremendously difficult to establish a minimally objective cut-off point between one function and another, given how interconnected and dependent they are. Article 1 of the CRPD (United Nations, 2006) groups human impairments into four categories: physical, mental, intellectual, or sensory impairments. The ICF (World Health Organization, 2001), on the other hand, divides human functions into eight groups: mental functions, sensory functions, and pain; voice and speech functions; functions of the cardiovascular, hematological, immunological, and respiratory systems; functions of the digestive, metabolic, endocrine systems; genitourinary and reproductive functions; neuromusculoskeletal and movement-related functions; functions of the skin and related structures. One might argue that this difference is due to a greater level of systemization and detail on the part of the ICF, and this is indeed the case (the aim of the UN Convention is not to list all the different impairments). The problem, however, lies not in the number of functions, but in the way they are classified. While the CRPD distinguishes between mental and intellectual functions, the ICF includes the latter among the former. For the sake of argument, I classify the wide range of bodily functions into three large groups: mental, physical, and sensory.

An individual’s degree of functional autonomy is related to the state of their mental (cognitive, psychological and emotional, awareness or memory-related, etc.), physical (motor functions, anatomical functions and structures, those related to voice and speech and, in general, all physiological functions of the human body), and sensory functions (visual, auditory, olfactory, gustatory, tactile, and pain-related). However, it is important to note that a person’s degree of functional autonomy will be made up of a combination of their capacities and the possibilities of exercising them provided by their environment. To put it very simply, capacity is merely the aptitude or skill a person has to perform a task or action. They are resources that are inherent to the individual, but which require the right external conditions to be exercised. The way people function is nearly always conditioned by their environment, which rarely plays a neutral role in the extent to which they realize their capacities. It is often rightly remarked that a lack of functional autonomy derives not only from the disabilities people have but also from disabling environments. We shall return to this matter below.

When the disease is chronic, patient autonomy is greater, but also more complex. It becomes more mundane, applying to more every day, and long-term cases. Autonomy goes beyond mere decision-making and becomes a *process* which is extended or *executed* over time. In simple terms, e*xecutive autonomy* may be defined as the capacity to implement the decision made and maintain it over time, in other words, to execute it. In the clinical sphere, this means that it involves the patient’s capacity to plan, sequence, and perform tasks related to the management of their chronic disease, especially those related to the planning and execution of treatment (Naik et al., 2009). Whereas functional autonomy relates to the material possibility of performing a task (e.g., getting dressed without help), in the case of executive autonomy, the essential aspect is the ability to keep to the course of action decided upon (e.g., quitting smoking). This element of autonomy was already implicitly suggested in some early bioethical works, such as the definition of the autonomous person given in the 1979 Belmont Report (National Commission for the Protection of Human Subjects of Biomedical and Behavioural Research, 1979) or the condition of intentionality which Faden and Beauchamp (1986) developed in their ethical theory of autonomous action. However, its importance is greater when we shift the perspective from acute care, where a plan of intervention and care is authorized by the patient and executed by the clinical team, to chronic care, where the patient authorizes that plan and then plays an essential role in implementing it.

Healthcare professionals tend to interpret patients’ non-compliance or abandonment of therapy as a conscious and autonomous refusal to follow their recommendations or as the result of deficient understanding of the nature of the disease or the proposed therapeutic regimen. However, some patients with chronic conditions may be capable of articulating a clear understanding of the treatment and be entirely convinced that they will adhere to it when they visit the doctor but then prove incapable of performing the required tasks in their everyday lives. Clinicians generally have little awareness of these impairments, especially those linked to executive capacities, and do not actively take this aspect into consideration when developing treatment plans. This incapacity is ethically and clinically significant, as the patient’s executive autonomy may be essential for effectively supervising and executing the treatment plan (Naik et al., 2009, p. 24). As well as the problem of non-adherence, the consequences of ignoring or understating the importance of executive autonomy can be “poorer health outcomes for patients, repeated hospitalizations, and frustrated clinicians” (Russell, 2009, p. 32).

Autonomy is extolled in individuals, but individuals are only autonomous with and thanks to others (Casado, 2014). Good care is the product of a dialog (Nys et al., 2007, p. 15). Rita Charon and other early advocates of the concept of narrative medicine have argued that communication between doctor and patient is the key to implementing a more humane model in medicine. In the last few decades, fields such as law, history, philosophy, anthropology, sociology, and politics have become aware of the importance of “narrative knowledge” (Charon, 2001, p. 1898). More recently, there has also been much talk of a “narrative shift” in bioethics, medicine, and nursing. The main purpose is to provide patients with better care, gain a deeper knowledge of their cases, understand them, and be closer to them (López de la vieja, 2013, p. 25). This cooperative model requires a certain “narrative competence” on the part of the practitioner, i.e., “the ability to acknowledge, absorb, interpret, and act on the stories and plights of others” (Charon, 2001, p. 1897). The doctor must be able to see beyond the “case” to be treated, in order to take in the whole-life situation of the individual (Gadamer, 1996, pp. 56–57).

The loss of balance experienced by a patient with a disease is not only a medical/biological fact; it is also a process linked to their life history and their relationship with others. The patient is no longer the same person as before. The individual becomes alienated and detached from their life story. This is where the idea of *narrative autonomy* comes in. This is the capacity that patients have to retain, understand, and communicate, coherently and understandably for others, both the circumstances of their present situation and the subjective, identitary, and cultural aspects that have characterized them during their lives and which may be of relevance when it comes to developing a suitable and respectful line of therapeutic action. Narratively autonomous patients are capable of integrating their decisions into a narrative that they can share with others, complementing the practitioner’s clinical record with a first-hand personal vision of their illness (Casado and Etxeberria, 2014). Such narratives are both subjective—after all, who is better placed to tell their life stories than patients themselves?—and intersubjective, since they require other people to be capable of recognizing and accepting the account they build of themselves. Merely being able to articulate a narrative is not a guarantee of the patient’s narrative autonomy. Narrators must be capable of exteriorizing their experience, communicating their intentions, and using the necessary agential capacities to interact with and influence other people, and to do this, they require a minimally coherent and intelligible story that matches the reality. Having narrative autonomy means being capable of participating in certain types of communicative interactions with others, and it requires fundamental concurrence on the most basic features of the reality shared by patient and audience (Schechtman, 1996, pp. 119–120).

Finally, *informative autonomy* involves patients’ ability to access and control their personal, intimate, private, and public information (Seoane, 2013, p. 31). Informative autonomy covers, *inter alia*, the personal management of clinical information, the right to communicate or protect such information, the doctor’s duty of confidentiality, and the skills required to communicate with others about the condition (Casado and Etxeberria, 2014, p. 54). Whereas in the decisional dimension, the information has an instrumental value and refers to all medical aspects (details about treatment, side effects, etc.) that the patient needs to know to make an informed decision, in the informative dimension the information has an intrinsic value and enables patients to decide for themselves when and under what conditions they disclose situations referring to their own life and health (Seoane, 2013, p. 31). Either through omission or ignorance, certain aspects of informative autonomy have yet to be integrated or consolidated in healthcare. Most theoretical studies and legal provisions to date have been written from a traditional perspective, focusing above all on the obligation to professional secrecy, patient privacy, and the confidentiality of the clinical documentation. However, a wider approach is needed that will also cover the most essential element of the informative dimension in the clinical field, i.e., all matters related to the protection, safekeeping, and management of personal data by the patient (Seoane, 2010, p. 64).

## Autonomies in Interaction

In legal/juridical constructs of autonomy, it seems logical to equate the concept with a certain psychological capacity that individuals require in order to make decisions and assume responsibilities. In these theories, the central aspect is individuals’ mental state, their transitory or permanent capacity to take responsibility for their actions. Christman (1989, pp. 5–6), for example, observes that the “psychological ability for self-government” is the common core of all conceptions of autonomy. He argues that features such as authenticity or self-determination, or notions of autonomy such as individual choice or political right, derive from this initial characteristic. However, in the medical domain this approach is insufficient. Patient autonomy has many different faces (Schermer, 2002) which would be excluded if one were only to cover mental or psychological aspects.

The different dimensions of patient autonomy should not be viewed as isolated realities, but rather as a continuum (Seoane, 2010, p. 63). Nonetheless, it may be helpful to address each one independently. Each dimension becomes especially visible at different moments or stages in the clinical-care process (when approving a medical operation, introducing a given treatment, managing information on a patient, dealing with people with physical disabilities or some degree of dementia, etc.). The duties and obligations they involve for healthcare practitioners and carers also vary (respect in some cases, restoration or promotion in others, etc.). As already discussed, autonomy entails considerably more than just decision-making by the patient and respecting that autonomy involves much more than simply presenting an informed consent form for signing. What is generically known as “patient autonomy” arises in different circumstances and in very different ways; some are well-established and traditional (such as decisional autonomy), but others have yet to be integrated or consolidated in the clinical relationship.

As stated earlier, the five manifestations of patient autonomy are not necessarily related or mutually dependent. Some patients enjoy only limited functional autonomy yet are decisionally, executively, or narratively autonomous. In other cases, poor executive autonomy may be found with no other significant autonomous deficit. Other patients are capable of self-determination but are the object of a pact of silence, and so on. In all of these cases, we see a problem in the patient’s capacity for self-government, but only in one of the five elements that together make up their autonomy. However, a phenomenological examination shows that an impairment or damage to one aspect of a person’s capacity for self-government can strongly affect other aspects. The dimensions of patient autonomy can be seen as a connected net: if one element falls, it can drag down another or even all of the others. They therefore need to be studied separately, but also, as Naik et al. (2009) suggest, it is necessary to study the “biopsychosocial correlates” linking them, given that a major shortfall in one manifestation of autonomy may act as a limitation in other dimensions. If a person suffers brain damage as the result of an accident, different manifestations of their autonomy will be impacted. When patients lack any kind of information on their condition, they will be unable to decide and act freely. If a tetraplegic person does not have adequate resources to lead their life, many questions related to decisional or executive aspects will no longer be relevant, etc.

Let us take, for example, the connection between decisional and narrative autonomy. Some psychiatric patients (including those in hospital) with a distorted narrative autonomy have been found to be capable of making fairly uncomplex decisions regarding their treatment and other areas of their lives. However, in general, a person’s narrative capacity is what sustains and legitimates decision-making; patients will retain their decisional autonomy as long as they know how to frame their desires and decisions within a narrative that is coherent and intelligible for themselves and others. Patients’ decisional autonomy only makes sense if it is framed within a wider identitary and agential framework within which the individuals explain themselves and establish relations with others. To put it another way, a person will show capacity for complex decision-making (and there will therefore be an obligation to respect their wishes) to the extent that they manage to integrate what happens in their life into an autobiographical narrative which matches the reality and perceptions of those close to them.

**Figure 1:**
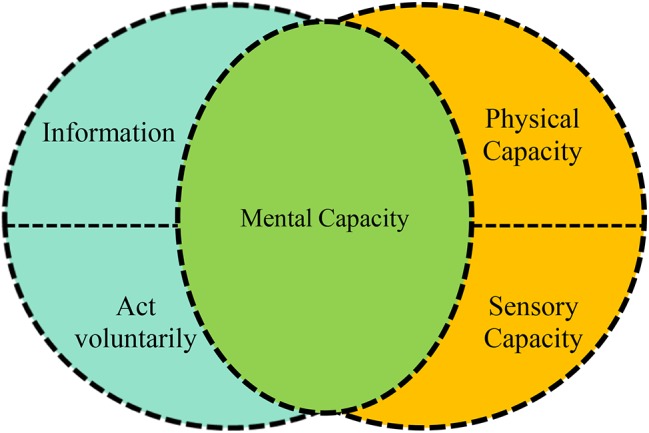
Relationship between decisional and functional autonomy.

Let us now look at the interaction between decisional autonomy and functional autonomy. Take the case of an individual who is entirely healthy and competent from a psychological point of view but who has suffered a medullar injury as a result of an occupational accident and requires rehabilitation to walk again. Such a person currently has a lack of autonomy even though their psychological ability for self-government is not impaired. In this case, the work of all the agents involved (nurses, doctors, family members, public institutions, etc.) must be geared not so much toward “respecting” their autonomy as “promoting” or “restoring” it. Let us now consider the opposite case, a patient with no significant physical or sensorial impairment but with a serious mental disorder. Here too, the patient has a lack of autonomy. However, whereas in the first case, the absence of autonomy was only functional and did not concern their capacity for decision-making (which needs to be respected just like that of any other patient); in the second case, the mental damage not only represents an impairment to their capacity for decision-making but also to other aspects of their autonomy. An important deficit in a person’s mental capacity will result in a diminishment of both their decisional and functional autonomy. We can therefore see that people’s mental capacity is the link between decisional and functional autonomy (see Figure 1).

**Figure 2:**
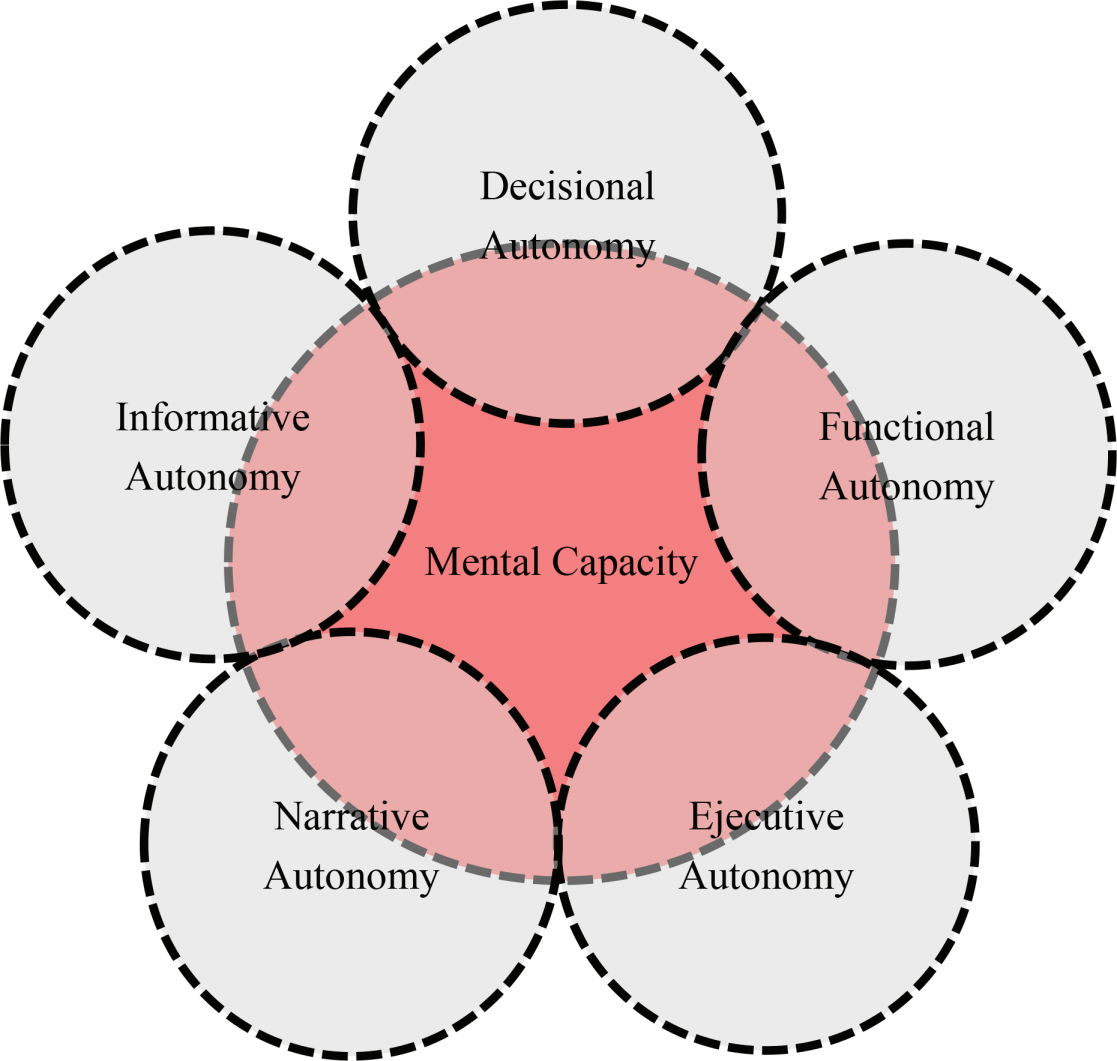
Mental capacity, necessary element of patient autonomy.

This link can be found in all dimensions of autonomy. Unlike physical, sensory, or executive capacity, in order for human autonomy to exist, there must always be some degree of mental capacity. An inability to walk or see usually entails a reduction in autonomy, but it does not necessarily mean an absence of other aspects of self-government. On the contrary, however, in cases of serious mental damage, no other dimension of autonomy is possible. It therefore follows that the individual’s mental capacity is a necessary but not in itself sufficient element of patient autonomy (Arrieta, 2016). It is a necessary element because all dimensions require a certain mental capacity on the patient’s part. Patients with very severe mental impairments are not capable of making decisions (decisional autonomy); of performing for themselves many tasks that a statistical majority of people can perform (functional autonomy); of keeping to a given treatment over time (executive autonomy); or of manifesting their communicative intentions in such a way as to mold the response of their audience (narrative autonomy). And clearly, we can also rule out any informative autonomy, which requires that patients be “capable” of controlling and managing their personal information. Yet mental capacity is not in itself sufficient because limited autonomy may be due to a mental disability but also to other factors: there are patients who are fully mentally capable but have problems with autonomy (see Figure 2).

To illustrate this thesis, let us return to the issue of psoriasis. The disease comes in many variants, ranging from miniscule marks on an elbow to the most severe forms, which affect over 90% of the body surface, and which cause serious and even fatal health problems for the patient. In most cases, having psoriasis does not involve a physical deterioration or limitation in a person’s functional, motor, or mental capacity. To put it simply, people who are diagnosed with psoriasis are perfectly capable of continuing with their normal everyday activities (except in the most severe cases or those with a major psychological impact). Yet many patients have difficulties in another aspect of their autonomy, namely their capacity to apply the treatment agreed upon with their medical professionals. In the following section, I shall examine in greater detail the interaction between decisional autonomy and executive autonomy. I aim to show that by working jointly on the two dimensions, it is possible to increase adherence to treatment in many chronic diseases.

## Decisional Autonomy, Executive Autonomy, and Psoriasis

Among the different clinical tools available for assessing the mental capacity of patients with a psychiatric or medical pathology, perhaps the most useful and effective is the MacCAT-T (MacArthur Competence Assessment Tool for Treatment) interview (Grisso and Appelbaum, 1998). This instrument has become “the benchmark protocol” for evaluating mental competence (Simón, 2008, p. 345). It is the most widely used tool around the world, enjoying the greatest empirical support (Pose, 2015, p. 82) and offering the greatest reliability to evaluators (Ventura et al., 2014). The interview assesses the patient’s degree of competence in decision-making in four psychological areas: (1) *expression of a choice* by means of verbal, written, or sign language. This is the first and most elementary skill; (2) *understanding* of information relevant to the decision to be made; (3) *appreciation*, adequate assessment of the patient’s specific situation; and (4) *reasoning*, capacity to develop a system of logical argument, to use the information the patient has understood and appreciated to arrive at a decision (Grisso and Appelbaum, 1998). Although other authors have since made their own contributions, these are still the four essential criteria when assessing patients’ clinical and psychiatric psychological capacity, and they have even begun to be used to construct simple decision-making algorithms (Simón, 2008, p. 338).

It is important to note that the score obtained from a MacCAT-T does not offer irrefutable and categorical proof of a patient’s general ability or inability to make decisions. As the authors themselves say (Grisso and Appelbaum, 1998), the scores obtained almost never form the basis for a definitive judgment of capacity. Unlike an individual’s weight or height, their decision-making capacity is not fixed and invariable, nor is it independent of human relations; rather it is the ability to understand and decide what is suitable for that individual depending on the context, the situation, and their state at any given moment in time. For this reason, the individual’s capacity must be assessed for each specific decision, not in overall and definitive terms; indeed, Grisso and Appelbaum themselves recommend reassessing capacity whenever necessary.

It is my belief that this clinical assessment of the mental capacity of patients with chronic diseases should be extended to include the capacity to adhere to an agreed treatment plan. Patients may be capable of engaging in a forthright deliberation on the planning and goals of the treatment but be physically, cognitively, or educationally incapable (sometimes without being aware of their inability) of carrying it out successfully. The degree of executive capacity patients show has, in part, a physiological or biological explanation. In areas such as neurology, psychology, and psychiatry, the concept of “executive control functions” is very commonly used. Executive functions are cognitive and psychological skills that allow the individual to perform tasks such as anticipating and setting goals, forming plans and programs, self-regulating tasks, and carrying them out efficiently. In essence, the executive functions are concerned with “directing” behavior toward an objective, of structuring it over time. This temporal structuring of conduct is performed through the coordination of three subordinate functions, the retrospective function (required for short-term memory), the prospective function (which essentially aids conduct planning), and the control and supervision function (which enables control of stimuli and internal and external influences that may affect conduct) (Fuster, 1980/2008). In a certain sense, the executive functions are “the brain’s brain” (Lopera, 2008).

The scientific literature confirms that the frontal lobes are the neurobiological base of the executive functions. Patients’ executive skills have a known anatomical substrate. They reside in a specific place in the brain, the prefrontal cortex. As a diagnostic criterion, this is of great clinical use, since it allows empirical studies to be performed to determine whether the patient has some problem or anomaly in that area. The possible alterations that may arise following an injury to the frontal lobe are very varied: they include cognitive, emotional, mnemic, motor, personality, and behavioral impairments. The reason for this wide variety of symptoms lies in the many higher functions governed from this lobe and the complexity of its associations with other cortical and subcortical areas of the brain (Rodríguez del Álamo et al., 2003, p. 605). Recent studies have identified associations between impairments in executive control functions and treatment self-management, performance, and outcomes of chronic medical and psychiatric conditions (Naik et al., 2009, p. 28). These studies provide important empirical evidence to support the idea that one of the requirements of proper care for chronic patients is not only to assess their decision-making capacity and respect their decisions but also to assess their capacity to carry out different tasks related to disease self-management.

For a detailed assessment of different aspects of executive functions, a broad repertoire of tests has been developed in the field of neuropsychology. Those most frequently used are the Wisconsin Card Sorting Test (WCST) and variations of the Tower of Hanoi (Tower of London, Tower of Seville, etc.). These tests require a certain degree of sophistication and are quite complex to apply and interpret. Consequently, abridged tests have been developed that are simpler and quicker to conduct. The oldest and perhaps best-known of these is the “Executive Interview,” also known as EXIT 25. Another very well-known tool is the “Frontal Behavioral Inventory” (FBI), a survey directed not at the patient, but at the carer or person in charge of looking after the patient. The aim of this test is to pick up on positive or negative changes in the patient’s conduct and personality. The “Frontal Assessment Battery at Bedside” (FAB) is also very widely used. It takes around 10 min to perform and explores the functions of the frontal lobes.

Clinical tools commonly used to assess decision-making capacity (such as the MacCAT-T) should be enhanced with others that assess executive capacity. Impairments in executive autonomy can occur independently of or in conjunction with impairments in decisional autonomy (Naik et al., 2009). The cognitive areas assessed by the MacCAT-T (understanding, appreciation, reasoning, and expression of choice) should be complemented with an assessment of the psychological and behavioral aspects covered by tools that assess executive functions, in order to obtain a more complete map of each chronic patient’s abilities for autonomy. In the case of frequent readmissions due to exacerbation of the disease, adverse effects of the medicine or other supposed markers of non-adherence, the doctor should consider whether the patient’s executive autonomy to administer the complex treatment plans and integrate them into their everyday life has deteriorated, either in isolation or in conjunction with impairments in their decisional autonomy. In short, effective treatment planning can be achieved through a dynamic and iterative process of identifying patients’ decisional and executive limitations and compensating for deficiencies in their executive capacity with appropriate clinical, family, and social support.

It is also important to remember that these tests or protocols are above all intended to assess executive deterioration in very elderly people, with some form of dementia or neurodegenerative disease. More than detecting and assessing executive functions, their primary role is to detect and assess *executive dysfunctions*. This being the case, it would be helpful if the perspective of these tools was to be broadened and if they were to be reworked to assess the capacity of patients who are seen to have difficulty implementing their own decisions regarding their health or care plan. Poor executive autonomy (in any patient, not only the elderly or those with dementia) may be the result of an impairment or disease (schizophrenia, Alzheimer’s disease, attention deficit disorder, depression, addiction, etc.); however, it may also be due to other emotional, educational, or cultural factors that are unrelated to the disease (or predate it). Indeed, weak executive autonomy is not always associated with an illness or impairment; it may also reveal a frequent condition which is very typical among humans. Many people—sick and healthy alike—at times lack the inner strength they need to master themselves and overcome the most immediate desires or urges that deflect them from what they consider to be a higher goal (in this specific case, properly adhering to the treatment). Since ancient times, all the most influential ethical constructions in western civilization have concerned themselves with this *akrasia* or weakness of will. In Book VII of his *Nicomachean Ethics* (one of the first philosophical treatises on the issue of continence and incontinence), Aristotle (2011) examined some very common cases in which the moral agent displays no consistency but rather an internal division. When the rational part wins out, the result is *enkrateia* (continence), but when the irrational part (desire) vanquishes, we have a case of *akrasia*, that is to say, an agent who has a moral understanding of right but is led by an opposing desire not to submit to it. However, Aristotle does not link *akrasia* to any disease. *Akrasia* or incontinence (“I see the better and approve it, but I follow the worse”, as Ovid puts it) extends beyond the clinical or medical field and is an essentially moral question. It is an example of human weakness of our tendency to passively follow an impulse rather than a deliberated option.

Just as everyone is characterized by being functionally different, there is also such a thing as “executive diversity”: each individual plans and implements decisions about their life and health in their own way, at their own pace, and there appears to be no rule determining what is executively normal or healthy and what is not. Thus, just as there is no disease involved in many cases of functional diversity nor is any disease involved in many cases of executive diversity. Each individual is unique and unrepeatable; an individual’s degree of executive autonomy will be the result of their pathology, their cognitive capacities, but also of their education and way of being in the world. Medics can treat patients with functional difficulties, cognitive barriers, or simply psychological features (untidiness, impulsivity, laziness, or excessive busyness, etc.) that hinder continuity between the decision they have taken at a given point in time in the doctor’s surgery and what needs to be done over a longer period.

Because adherence to topical treatment is a complex, multifactor issue with factors varying between patients, dermatologists should focus on determining each patient’s individual adherence barriers to achieve good treatment outcomes (Choi et al., 2017). Factors influencing adherence include patient-specific characteristics, disease-related characteristics, treatment satisfaction, cosmetic acceptability, and the complexity of treatment protocols (Kuehl and Shear, 2018). At the same time, the role of the patient/physician relationship is a key issue in the management of lifelong, chronic conditions such as psoriasis. Patients want more information on psoriasis, fast treatments, clear expectations from the onset of therapy, and recognition of the emotional burden (Uhlenhake et al., 2010). Therefore, the better doctors know their patients (psychological profile; personal circumstances and motivation for combating the disease; time they have or will have available for administering the therapy; expectations and experiences with other treatments), the greater their chances of getting the treatment plan right and ensuring better adhesion. In this regard, new topical therapeutic options need to offer a combination of higher efficacy and better patient acceptability, including easier application, to reduce treatment burden and enhance patient adherence. Recent studies report that cosmetic acceptability is a key contributor to adherence. Topical spray foam vehicles are innovative alternatives to creams and ointments. Well-designed spray foam vehicles are easily spread over large areas of the skin, while importantly not leaving a greasy or oily film on the skin after application (Kuehl and Shear, 2018).

## Discussion: Patient Autonomy and Identity

Any consideration of autonomy must necessarily take into consideration the way in which agents interact with the environment in which they live. The autonomy of any living being must be accompanied by a certain context or environment which is conducive to the exercise of that autonomy. An individual’s degree of autonomy is related, on the one hand, to their capacity to perform different human activities, such as making a rational and conscious decision, managing their time or even pouring themselves a glass of water, and, on the other, to the range of possibilities offered them by the environment to develop or exploit these skills. Moreover, the development and exercise of the capacities enabling human autonomy are profoundly social. Only in a context of social interaction and mutual recognition, individuals can construct and develop their autonomy.

This social conception of human autonomy has been driven in recent decades by feminist philosophy and moral psychology. In the 1970s, feminist praised the ideal of autonomy and extolled its liberatory potential for women. In the 1980s, this view was challenged by other feminists who rejected the ideal of autonomy as it had traditionally been conceived. They regarded the notion of autonomy with suspicion because it was thought to presuppose a conception of the person as “atomistic,” as ideally self-sufficient, as operating in a vacuum unaffected by social relationships, or as an abstract reasoner stripped of distorting influences such as emotions. The 1990s witnessed a renewed feminist interest in autonomy but as *relationally* conceived (Friedman, 1997, p. 40; Stoljar, 2018). The term “relational autonomy” does not refer to a single unified conception of autonomy but is rather an umbrella term, designating a range of related perspectives. These perspectives are premised on a shared conviction, the conviction that “persons are socially embedded and that agents’ identities are formed within the context of social relationships and shaped by a complex of intersecting social determinants, such as race, class, gender, and ethnicity” (Mackenzie and Stoljar, 2000, p. 4).

Viewed in this way, autonomy reflects the capacity to perform tasks depending on the individual’s environment. In its most basic definition, capacity means the “ability to do” (being able to breathe, able to reason, able to walk); this requires from the agent both the material possibility of performing an activity or task and the skill or ability to carry it out. The first condition can be measured in absolute terms: one either has or does not have the possibility of doing something. It is, one might say, an internal or external imposition or limit, regardless of the agents’ volition or predisposition or the society in which they live. Human beings are incapable, by themselves, of flying or breathing under water. However, the second condition is more gradual and flexible and may be manipulated by human interaction and technical and technological advances. People who do not speak English will be incapable of understanding this text, although they have the possibility of doing so, either by learning the language or by using a translation tool (Arrieta, 2016). The individual is a “spectrum of ability” (*faisceau du pouvoir faire*) (Ricoeur, 2008, p. 72) which is manifested in multiple domains of the human: power to say, power to act on the course of events and to influence the other players in the action, and power to bring one’s own life together in an intelligible and acceptable narrative. The notion of capacity constitutes the ultimate reference of moral respect and recognition of the human as a holder of rights, and it is closely associated with the notion of personal or collective identity (Ricoeur, 1997, pp. 28–29).

However, in extensive areas of healthcare ethics, there is a tendency to use a notion of autonomy that has been idealized, as if it corresponded to the needs of mature, healthy, and self-sufficient citizens who make decisions independently, consciously, and rationally. Insofar as they restrict themselves to mental or psychological capacity, models of autonomy taken from the philosophical/legal tradition are deficient for constructing a patient’s autonomy, especially when applied in the context of chronic disease and long-term care. Here, we need to establish a model of *autonomy within illness* (Casado and Etxeberria, 2014), which is different from that conceived and enjoyed by healthy individuals. This reconceptualization requires, *inter alia*, an awareness of discoveries in neuroscience and the cognitive sciences and an emphasis on the relational nature of autonomy, two lines of work whose findings largely coincide. While physicians such as Cassell (2010) and Tauber (2005, 2011) observe that we cannot apply a concept of autonomy to the healthcare relationship that is more characteristic of healthy individuals than sick ones, recent works in the field of neuroscience suggest that even the autonomy of healthy subjects does not match the standard model. Felsen and Reiner (2011) show that human brains are capable of the hierarchical control required for reflective thought, but that decisions conventionally perceived as autonomous may not be rational with respect to the deliberative process itself, and are rarely free from covert external influences. If the capacity for autonomy of healthy individuals needs to be redefined in order to align our moral values with neuroscientific naturalism, what about patient autonomy? It is even more complex and precarious than the autonomy assumed by the standard model (Moreno and Casado, 2011).

In our research group (“IAS Research - Center for Life, Mind & Society”), we view autonomy as the preservation of an identity over time through interaction with the environment. Rather than just a capacity for self-government, we see autonomy as the way in which certain complex systems manage to maintain a precarious identity through the generation of actions that ensure this continuance. What the agent *does* (conduct) is ultimately related to what the agent is (organization) and vice versa (Barandiaran and Moreno, 2006). From this perspective, the most important aspect is the mutual relationship which exists between maintaining the identity of an autonomous agent—in this context, a human—and that individual’s performance in the environment (Moreno and Casado, 2011, p. 54). Hence, in defining the identity of the autonomous agent, it is essential to take into account both the constitutive aspects (internal organization of the system) and the interactive aspects (relationship with the environment) (Etxeberria and Casado, 2008, p. 13). Indeed, human beings are constitutively interactive, and inversely, interaction makes us human and moral beings. Interaction with the environment is a constitutive element in the emergence of the social and cognitive capacities of living systems.

Although they do not explicitly distinguish between functional and executive autonomy, Casado and Etxeberria (2014) have argued that the different elements of patients’ autonomy can be ordered on an axis that is related to the tension between their constitutive aspects (in the sense that they are properties of the patients *vis-à-vis* themselves) and their interactive aspects (the properties of the patients *vis-à-vis* others, such as medical practitioners and society at large). Decisional, executive, and functional autonomies are constitutive in nature because they mostly emerge from the patient’s personal qualities. However, the other two kinds of autonomy, narrative and informative, are interactive in nature; their exercise depends to a large extent on social and environmental factors and on the role played by people from the patient’s environment. Thus, patients’ power to decide for themselves when and under what conditions they choose to disclose situations related to their own lives and health (informative autonomy) will be subject to the cultural and legal modes of operating of the community in which they live. In a society that attaches little importance to the intimacy and privacy of its members, people will have little informative autonomy, however much they might desire it (Arrieta, 2016).

At the same time, any ethical judgment on a given situation depends not only on the decision-making of its participants but also on their mutual interaction (Colombetti and Torrance, 2009). This leads us to think that the patient’s autonomy emerges as a consequence of the new identity they assume as a patient based on their interaction with practitioners, family, and society in general (Arrieta and Casado, 2014). This can be seen very clearly in the case of the other interactive autonomy, narrative autonomy. Human disease can no longer be seen as an isolated and “objective” fact, far removed from the “story” of the individual who suffers it (Arrieta, 2012). Elsewhere, I have argued (Arrieta, 2016) that the prevailing concept of autonomy both in medicine and in clinical ethics is more closely linked to the professional vision (*disease*), than to the social vision (*sickness*) or the personal vision (*illness*). An essential feature of modern western medicine is that it has prioritized the vision of the patient as an object rather than a subject. Seduced by a scientific ethos, modern medicine has tended to address the disease rather than the patient. To be fair, evidence-based medicine has obtained good results, but many specialists believe it has also led to a decline in the quality of care and the human quality that should characterize the art of curing. Instead, they advocate “patient-centered medicine,” which addresses in equal measure the emotional, psychological, and social aspects of the affliction of individuals requesting attention. In addition to the objectifying and third-person account that is characteristic of natural science, we need to bring in the subjective first-person account of the individual who experiences and feels the illness. When a disease is more or less chronic, no curative action of any quality can be provided without an understanding of what the disease is doing to patients’ self-esteem and the content or narrative focus of their lives.

We are, to a very large extent, the stories of our lives. The way in which the disease affects us depends on the way in which the sickness alters our stories (Brody, 2003, p. 269). Moreover, for many people, the pain, suffering, or incapacity resulting from different adverse situations (a serious disease or accident, bereavement, etc.) generate additional suffering because they burst in upon them dramatically and unexpectedly, because they entail a clear disruption of their present situation, and because they mark a “before and after” in their lives. In similar situations, we find ourselves intellectually and practically disconcerted because, for some time at least, we do not know where to place these events in our life story. Over recent decades, many philosophers have argued that identity and human life are constituted narratively (MacIntyre, 1981/2007; Schechtman, 1996, 2012; Ricoeur, 1997, 2008; Gracia, 2004). The profound importance of narratives lies in the fact that they configure us in moral and identitary terms, for the fundamental reason that life has a narrative structure. People constitute their identity through the development of autobiographical narratives, explaining the circumstances that happen to them in their lives through accounts or stories that make sense of them. Unlike other living beings, narrative in humans is an organizing principle of our lives and the lens through which we filter our experience and plan for actions (Schechtman, 1996, p. 113). Individuals constitute themselves as people by thinking of themselves as persistent subjects who have had experiences in the past and will continue to have experiences in the future. The unit of identity is a narrative, the “storyline” we attribute to our lives: we constitute ourselves as people through an understanding of our lives as narratives in the form of a person’s life story. This need to forge our own story, to “tell ourselves,” is especially visible in the field of healthcare and human disease; it is never an isolated event that can be separated from the context of the life and story of the individual and community who suffer it (Gracia, 1991/2007).

Disease (and the pain and/or suffering that accompany it) is the effective cause that triggers the beginning of a care relationship. The disease disrupts the agent’s relationship with their own body; it alters individuality and therefore our understanding of and the relevance we attach to autonomy. Pain and suffering are in themselves a source of reduced capacity for self-government. When we get sick, we cannot function normally as individuals because our capacity to be a “self” is endangered. For affected individuals, entering a state of illness involves a series of transformations in their bodies, their subjectivity, and their physical, social, and cultural worlds (Carel, 2008). Human existence is embodied and defined by perceptual experience and thus, any alteration in the body and in people’s physical, perceptual, or behavioral possibilities entails a transformation in their identity and their capacity for self-government (Meyers, 2005).

The physical, psychological, and social effects of psoriasis can represent a major setback to the mood and quality of life of not only patients themselves but also their next of kin. In diseases of this type, it is not unusual to find a mismatch between how patients see themselves and how others see them. Trying to correct that distortion means emphasizing aspects of the patient’s narrative autonomy. For some of those affected (especially in mild cases of the disease, when it does not notably disrupt their everyday activity and way of life), it is little more than an annoyance, another symptom of their imperfect reality. Many others, however, are greatly affected by the cultural norms surrounding image. They suffer greatly from having a “visible” skin disease which has traditionally had a very bad press (in former times it was erroneously associated with leprosy) and which weakens, frustrates, embarrasses, alienates, and stigmatizes them, in many cases making psychological treatment necessary. It is estimated that at least 100 million individuals are affected worldwide, and this condition is becoming more common, since an apparent upward trend is observed in several countries (World Health Organization, 2016). Psoriasis is one of the most frequent reasons for dermatological consultation and one of the chronic diseases with the lowest rate of adherence to treatment. While there are many reasons for this phenomenon, assessing the executive capacity of each patient and acting accordingly might help increase adherence to treatment, resulting in an improvement in the living conditions of people with the condition.

## Data Availability

All datasets generated for this study are included in the manuscript and/or the supplementary files.

## Author Contributions

The author confirms being the sole contributor of this work and has approved it for publication.

### Conflict of Interest Statement

The author declares that the research was conducted in the absence of any commercial or financial relationships that could be construed as a potential conflict of interest.

The handling Editor declared a shared affiliation, though no other collaboration, with the author IA.
